# Barriers to and Facilitators for Teachers’ Wellbeing

**DOI:** 10.3389/fpsyg.2022.867433

**Published:** 2022-05-23

**Authors:** Cassandra A. Gearhart, Madison Blaydes, Christopher J. McCarthy

**Affiliations:** Department of Educational Psychology, The University of Texas at Austin, Austin, TX, United States

**Keywords:** teacher wellbeing, public school teachers, stress management, barriers, facilitators, organizational level change, teacher stress

## Abstract

Teaching is widely recognized as a stressful profession, which has been connected to burnout and high turnover of qualified teachers. Despite increasing attention on teacher wellbeing, stress management interventions are often underutilized and demonstrate small effect sizes, and research on teachers’ informal stress management practices and desired resources is limited. It is likely that formal and informal intervention effectiveness is limited by teachers’ ability to access existing resources and navigate the complex educational systems they inhabit. The study explored the barriers to and facilitators for teachers’ engagement in formal and informal stress management interventions and desired resources across socioecological levels. Thirty-two teachers participated across four focus groups. Inductive thematic analysis was used to identify relevant themes. Personal barriers (e.g., guilt about self-prioritization), environmental barriers (e.g., mixed messages about self-care), and improved campus resources (e.g., scheduled opportunities to destress) were common themes. Recommendations for supporting teachers’ wellbeing include self-care affirming messages from peers and administrators, campus- and district-level changes to remove logistical barriers to stress management, and increased connectedness among campus community members.

## Introduction

Teachers often find fulfillment, joy, and value in their profession (Schutz, 2014). Teachers are motivated by the sense that they make a difference in children’s lives and in society as a whole (Bakar et al., 2014). These experiences contribute to job satisfaction and ultimately occupational wellbeing. Teacher wellbeing in the workplace has been difficult to define. A recent empirical attempt by Fox (2021) produced a four-component definition with psychological and social elements. These four components include teacher efficacy (i.e., teachers’ beliefs in their teaching abilities), teacher disposition (i.e., qualities and characteristics of the teacher), school connectedness (i.e., teachers’ relationships with others in the school), and job specific stress (i.e., experiences that may hinder wellbeing when disproportionate to the three positive elements of teacher wellbeing). This definition is substantiated by the current teacher wellbeing literature which states manageable stress is a key component of wellbeing (Ouellette et al., 2018; McCarthy et al., 2019), alongside social support (Ferguson et al., 2017) and personal resources (Jennings et al., 2013). Teachers are unfortunately some of the most stressed workers, with nearly half of educators reporting high daily work stress (Gallup, 2014).

High teacher stress can have negative personal and professional implications. Teachers’ high stress is associated with physical and mental health problems (Shernoff et al., 2011), increased intention to leave the field, and attrition (Ryan et al., 2017; McCarthy et al., 2019). Student wellbeing and achievement are also impacted when teachers struggle (Oberle and Schonert-Reichl, 2016; Herman et al., 2018). To further complicate the issue, teachers face a unique constellation of demands within their work environment: low student motivation, challenges disciplining students (Skaalvik and Skaalvik, 2017), burdensome workloads (Shernoff et al., 2011), and high-stakes testing (Lever et al., 2017) to name a few. There is clearly a need to address high stress experienced by teachers.

Efforts to reduce teacher stress often include stress management intervention (SMI), which intend to minimize stress through activities, programs, or organizational changes (Richardson and Rothstein, 2008; Lever et al., 2017). Interventions and policy changes can occur across socioecological levels, including individual change, individual-organizational interface change, and organizational change (Greenberg et al., 2016). Efforts to address teacher stress typically involve individual or individual-organizational interventions (Greenberg et al., 2016).

On the individual level, researchers and practitioners have implemented a variety of stress management programs, most designed to teach coping skills (e.g., mindfulness, cognitive reappraisal). These programs show modest improvements on burnout: small effects on workplace emotional exhaustion and sense of accomplishment and no effects on professional detachment with teachers (Iancu et al., 2017). In other professions (e.g., business), these programs typically yield medium to large effect sizes on similar measures of wellbeing (Richardson and Rothstein, 2008). Perhaps teachers’ highly structured workplace limits engagement in and benefits from individual change interventions. Also common are individual-organizational SMIs.

Individual-organizational stress reducing efforts in education include mentorship programs for early career teachers (Ingersoll and Strong, 2011) and reimagining teacher evaluation processes (Anderson et al., 2019). These interface changes are not yet standard practice but have shown promise for reducing workplace stress (Ingersoll and Strong, 2011). Lastly, change at the organizational level has yet to truly be enacted and evaluated in education settings (Greenberg et al., 2016). Organizational interventions aim to prevent stressful workplace conditions in the first place. Open communication, reimagining job structure, and improved training have been studied in other fields, but these interventions have yet to gain traction in education (Tetrick and Winslow, 2015). Greenberg et al. (2016) call for continued basic research on teacher stress interventions across levels.

An emerging topic in the teacher stress literature examines *informal* stress management among teachers. Informal stress interventions may include self-care practices (e.g., exercise, meditation) and social interaction (e.g., meals with colleagues, unofficial mentoring). Kelly (2021) examined teachers’ existing self-care strategies and found teachers use a variety of techniques, like seeking out friends and family for emotional support, reading books to promote intellectual wellbeing, and utilizing mentorship relationships for professional support. However, engagement in these activities may be hindered by barriers such as inflexible thinking, limited energy when stressed, and limited time (Barton, 2021; Kelly, 2021). Surprisingly, teachers’ experiences of formal and informal SMIs are rarely, if at all, considered together. Neither are the barriers prohibiting teachers’ management of occupational stress.

In the field of public health, access to intervention and care is viewed as prerequisite for health improvement (Shaw, 2012). Penchansky (1977) defined access as the necessary fit between the intervention characteristics (and contexts) and the needs of the intended population for initial and sustained engagement with health promotion systems. Yet, these authors found little to no attention paid to how SMIs are accessed by teachers. This study aims to fill this gap by exploring teachers’ experiences of barriers to and facilitators for engagement in formal and informal SMIs. To further contextualize SMI barriers and facilitators, the socioecological changes teachers view as necessary to improve SMI engagement and occupational wellbeing are discussed.

## Materials and Methods

Inductive thematic analysis, a process akin to grounded theory (Glaser, 1998), was employed to explore teachers’ discussions of stress management. Consistent with Glaser’s formulation of grounded theory, the current study is situated in a post-positivist paradigm. This means we viewed the experiences and perceptions shared by teacher participants in this study as reflective of the experiences shared among teachers more generally, while also being a product of ever-changing contexts. From this perspective, it is appropriate to describe the contexts surrounding and within focus groups, such as the local COVID-19-related regulations and frequency at which topics were discussed, as this information can shed light on how teachers experienced the conditions of their work-life at the time of data collection. The following sections detail the procedures for data collection and analysis. All procedures were approved by the Institutional Review Board at the University of Texas at Austin.

### Focus Group Format and Procedure

Focus groups were chosen for data collection as they allow participants to react and respond to one another thus generating a variety of responses, which aligns with the study goal of uncovering the breadth of wellbeing barriers and facilitators. Focus groups were conducted in partnership with a community mental health clinic located in schools. Clinic staff and school counselors facilitated scheduling and advertising for the focus groups. Groups were facilitated by the first author, a doctoral student in an American Psychology Association-accredited counseling psychology program, and assisted by a fourth-year undergraduate student majoring in human ecology. A total of four focus groups were conducted; two at elementary schools, one at a middle school, and one at a high school. All four schools belonged to the same large public school district. The first two focus groups (one at an elementary school and one at a middle school) were conducted in person just prior to local government shutdowns due to the novel COVID-19 virus (i.e., early March 2020). The last two groups (one elementary and one high school) were conducted virtually during shelter-in-place orders in April 2020. These two groups were conducted *via* Zoom video conferences during the school day. The number of teachers who chose to participate did not substantially vary between the two focus group formats [*x*^2^ (3, *N* = 32) = 0.75, *p* = 0.86].

Participant recruitment was consistent across all focus groups. Campus-wide emails were sent advertising the topics of discussion, participation incentives (i.e., $15 Target gift card and snacks for in-person focus groups), and assuring confidentiality. Interested teachers responded to the first author; no respondents were denied participation. Reminder emails were sent 2 days prior to and the day of focus groups. Some procedures had to change when focus groups went virtual; these differences are described next.

#### Focus Group Protocol

A 1-h, semi-structured protocol was followed for all focus groups. The complete protocol covered teachers’ sources of stress, coping strategies, available supports, barriers to and facilitators for resource use, and desired resources. The facilitator overviewed the generative goal of these focus groups, set norms for voicing alternative views, and communicated the importance for respecting confidentiality. Focus group questions most relevant to the current study included (1) What kinds of support services are currently available for stress management?, (2) What are the barriers to accessing these resources, (3) What facilitates use of these resources?, and (4) What are some other teacher stress management resources you think would be helpful for teachers at your school? It became clear during the first focus group that discussions needed to include a broader definition of stress management, as teacher often discussed individual self-care practices alongside formal campus resources and system-level policies. Probes were added across all focus groups to allow for a wider range of discussion. Teachers were encouraged to build on one another’s thoughts and help guide the discussion. Minimal redirection was needed.

#### In-Person Focus Group Procedures

Paper research consent forms were provided. Major points were covered then teachers were provided time to read over the form and ask questions. Signed consent forms were collected. Teachers had the option to provide work history in education (e.g., years teaching) and demographic information (e.g., race, gender) on tablets during the first 15 min of each group. A semi-structured protocol was then followed (see Focus Group Protocol below). Participants were given $15 gift cards for their participation.

#### Virtual Focus Group Procedures

A Qualtrics survey was used to obtain written consent to participate in research the day prior to focus groups. Teaching history and basic demographic information was also collected in this survey. Focus groups were conducted *via* Zoom.us. Meeting information, including password, were provided to participants *via* email. The second author verified all participants had completed the consent survey before the focus group commenced. Participants received digital $15 gift cards within 24 h of focus group participation.

### Participants

A total of 32 teachers participated across the four focus groups. To better understand the variety of perspectives across teaching faculty, recruitment was open to any staff member considered a member of school faculty. Nearly all grade levels, specializations, and educational roles were represented (i.e., prekindergarten through twelfth grade, special education, core and elective subjects, assistant teachers, librarians). Members of administration and non-faculty staff were excluded from participation in the current study. Of the 32 participants, 27 opted to provide demographic information. This information is reported in Table 1.

**TABLE 1:** Participant demographics.

Factor	Live Oak Elementary	Ash Middle	Mesquite High	Juniper Elementary	Overall
Focus group format	In-person	In-person	Virtual	Virtual	-
Number of participants	10	8	7	7	32
Number of participants who provided demographics	7	6	7	7	27
Average teaching experience (years)	18.6	17.5	9.6	19.1	16.13
Average age (years)	51.3	45.3	41.4	50.1	47.1
Gender (%)					
Male	0	16.7	14.3	0	7.4
Female	100	83.3	85.7	100	92.6
Race/ethnicity (%)					
White	14.3	83.3	42.9	71.4	51.9
Hispanic/Latinx	71.4	16.7	14.3	14.3	29.6
African or African-American	14.3	0	0	0	3.70
Asian or Asian-American	0	0	14.3	0	3.70
Biracial or multiracial	0	0	28.6	14.3	11.1

Teacher demographic information is provided by focus groups and across the sample. Theme and subtheme frequencies indicate the number of excerpts in which a specific theme appeared. Excerpts were created within transcripts to capture complete thoughts and provide consistent data segments for coding.

### Campus Characteristics

The two elementary schools, one middle school, and one high school included in the study were from the same independent public school district in the United States Southwest. Each school was assigned a pseudonym prior to analysis: Live Oak Elementary, Ash Middle, Mesquite High, and Juniper Elementary. All four schools were primarily populated with Hispanic/Latinx (25.7–73.0%) and White (14.3–54.8%) students, and Hispanic/Latinx (16.4–41.2%) and White (54.7–71.6%) teachers. Focus group participants reflected the distribution of early career teachers, racial and ethnic makeup within their respective schools. The percentage of economically disadvantaged students varied substantially between schools (19.9–72.7%), with Juniper elementary school qualifying as a Title 1 school for the 2019–2020 school year.

### Data Analysis

Inductive thematic analysis unfolded in three stages: data preparation, codebook creation, excerpting, and code application. These processes are described next.

#### Data Collection and Preparation

All audio files underwent transcription. For in-person focus groups, audio recorders were used to capture audio data. Audio files were stored in an encrypted, cloud-based storage platform prior to and during professional transcription. For virtual focus groups, meetings were recorded through Zoom.us. Autogenerated meeting transcripts were checked against the audio recording, and updated as necessary. Encrypted, cloud-based storage through Zoom.us was used throughout the process. Microsoft Word documents were used for transcripts. Names, places, and other identifying information were then redacted. Audio files and recorded meetings were permanently deleted after transcription. Narratives were then housed on Dedoose.com, a cloud-based qualitative analysis platform.

#### Substantive Coding and Codebook Creation

In the first phase of analysis, data-derived codes were taken from the first two focus group transcripts. In preparation for this process, two coders, the first author and an undergraduate student, shared expectations and biases they felt might sway the sorting process. This awareness allowed for an open conversation of bias throughout the coding process. Each coder then read through a transcript and summarized statements in a word or two. The goal in this phase was to create codes without conceptualizing teachers’ statements. Coders’ arising thoughts were noted and used during code sorting to further refine research questions, akin to theoretical memos in grounded theory (Glaser, 1998).

Data-derived codes were transferred to Mac’s Stickies application. Each substantive code received a sticky note. Coders then met virtually to sort codes thematically. This process yielded five preliminary themes that were then compared to authors’ process notes and existing theory. This process of constant comparison between existing data, ongoing notes, and theory continued through multiple iterations of induction and deduction until data-driven themes and subthemes took shape. The resulting themes and subthemes were defined and hierarchically organized into a codebook.

#### Excerpt Creation and Code Application

In the next stage of analysis, transcripts were excerpted into sections of text that captured teachers’ thoughts on sources of stress, coping strategies, stress management barriers or facilitators, or desired resources by the first author. These excerpts then served as consistent pieces of text for coding. Two coders, the second author, a doctoral student, and an undergraduate student, independently applied codes to excerpts then met virtually to discuss discrepancies and omissions. A consensus approach was used to determine final codes. When the two initial coders could not come to consensus, a third member of the research team, the first author, would join the discussion and make the final determination. This procedure was applied across coding phases, which included (a) pilot testing the initial codebook, (b) adjusting the coding process into two stages, and (c) verifying final codes. Each phase is described below.

The initial codebook was pilot tested on the Live Oak Elementary and Mesquite High excerpts. During code comparison meetings, revisions were made to the codebook based on patterns of disagreement and confusion. Revisions included the addition of child codes not previously captured and further dividing commonly occurring codes. The final codebook was then applied to the remaining two focus group transcripts with minor adjustments along the way. The coding procedure shifted halfway through due to inadequate interrater reliability.

Cohen’s kappa was run to assess interrater reliability, or the clarity with which emergent themes represent the data (McHugh, 2012). This is done by calculating the level of agreement between independently assigned codes. Cohen’s kappa also takes into account random agreement, or code agreement for reasons due to chance selection. Interrater was run throughout the coding process.

Early on in the coding process, moderate agreement was obtained when the complete codebook was applied to the Juniper Elementary transcript (κ = 0.66; McHugh, 2012). Given the complexity of the codebook (i.e., over 70 codes across four themes), the coding process was simplified to better assess the ability of the codebook and coders to distinguish between subthemes within major themes. To do this, coders independently determined whether excerpts discussed one or more of the major themes (i.e., stressors, coping mechanisms, barriers/facilitators to stress management, and desired resources). This was done for the last transcript, Ash Middle. Disagreements were discussed and final parent codes applied. Next, coders followed the same process to apply child codes according to previously determined parent codes. Interrater reliability at this level was almost perfect (κ = 0.91; McHugh, 2012).

Lastly, all final codes were reviewed for consistency. Possible inconsistencies were highlighted then discussed as a group until consensus was reached. Final code frequencies were calculated (see Table 2). Two of the four major themes are included in this study: stress management barriers/facilitators and desired resources.

**TABLE 2:** Code frequencies.

Code	Live Oak Elementary	Ash Middle	Mesquite High	Juniper Elementary	Total
Stress Management Barriers and Facilitators	46	59	41	23	169
Personal factors	15	25	8	12	60
Self-prioritization Beliefs	15	20	5	10	50
Barriers	15	15	5	10	45
Facilitators	0	5	0	0	5
Awareness	0	5	3	2	10
Barriers	0	3	2	2	7
Facilitators	0	2	1	0	3
Environmental Factors	29	33	31	10	103
Social Climate	6	9	7	1	23
Barriers	5	7	3	0	15
Facilitators	1	2	4	1	8
Administrative Factors	9	9	2	3	23
Barriers	7	6	0	2	15
Facilitators	2	3	2	1	8
District Factors	1	5	1	3	10
Barriers	1	4	1	1	7
Facilitators	0	1	0	2	3
Service Factors	8	5	11	3	27
Barriers	5	3	7	1	16
Facilitators	3	2	4	2	11
Campus Facilities	5	5	10	0	20
Barriers	5	3	7	0	15
Facilitators	0	2	3	0	5
Behavioral Factors	2	1	2	1	6
Barriers	2	1	1	1	5
Facilitators	0	0	1	0	1
Desired Resources	28	21	27	24	100
Individual	0	2	0	4	6
Autonomy	0	2	0	4	6
Campus	25	15	27	20	87
Colleague Support	5	3	3	4	15
Administrative Support	10	8	5	16	39
Parent Support	0	2	0	0	2
Facility Resources	10	2	19	0	31
District	2	4	0	0	6
Calendar Changes	1	4	0	0	5
Community Partnerships	1	0	0	0	1
Community	1	0	0	0	1

## Findings and Discussion

The following results were collected at the onset of the COVID-19 pandemic in spring 2020. Two focus groups were conducted in-person just weeks prior to shelter-in-place orders and the following two focus groups were conducted while virtual teaching was occurring. As noted in the methods, participation did not significantly differ between modalities. However, differences were found in overall theme frequencies. Barrier and facilitating factors were discussed more than would be expected during the Ash Middle school focus group and less than expected during the Juniper Elementary group [*x*^2^ (3, *N* = 32) = 15.78, *p* = 0.001]. These differences may have less to do with focus group mode and more to do with administrative differences between schools. The majority of teacher participants at Ash Middle felt supported by their administrators and provided a wide range of other environmental barriers to and facilitators for SMI engagement, whereas the teacher participants at Juniper Elementary spoke extensively about changes they felt were necessary at the school administration level and spent less time exploring barriers and facilitators. Overall, the frequency at which teachers discussed desired resources did not differ between focus groups. Next, the theories used to ground findings [i.e., Bandura’s (1986) triadic reciprocal causation model and McLeroy et al.’s (1988) ecological model of health behavior] and emergent themes’ relevance to SMI access are discussed.

### Teachers’ Experiences of Stress Management Barriers and Facilitators

**FIGURE 1:**
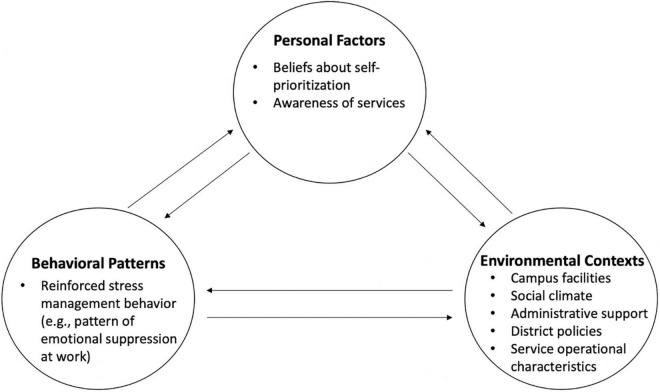
This figure depicts the triadic reciprocal causation model, a foundational aspect of social cognitive theory (Bandura, 1986), with emergent themes for the current study. Model elements together shape educators’ ability in meaningful stress management.

To address the first and second goals of this study, factors that hinder and support teachers’ management of stress were identified. Bandura’s (1986) social-cognitive theory felt particularly relevant for organizing and understanding emergent themes. The triadic reciprocal causation model, of social-cognitive theory, was used to group ideas: (a) personal factors, such as experiences of guilt over prioritizing one’s self and lack of awareness of resources; (b) environmental factors, such as unconducive campus facilities, social stigma, unsupportive administration, incompatible district policies, and inconvenient service characteristics; and (c) behavioral patterns, such as emotional suppression and reinforced boundary setting (see Figure 1 for a visual representation). Bandura’s emphasis on the complex, bidirectional relationships fit well with the often entangled personal (e.g., feeling pressed for time) and environmental (e.g., unclear administrator expectations) experiences described by teachers. Themes within these areas are described next.

#### Personal Barriers to Stress Management

Teachers frequently discussed the ways thoughts, feelings, and lack of resource awareness hindered their ability to manage stress, or personal barriers. Feelings of guilt about prioritizing personal needs over others’ needs and navigating time pressures were common and often overlapped, which led to their grouping under self-prioritization beliefs. The self-prioritization beliefs barrier was the most frequently discussed of all barrier themes. Conversations about guilt included taking time off when ill, losing time with family, and utilizing high demand resources: “That guilt of ‘I’m using a [therapy] slot,’ a time slot that maybe a kid [student] could use and I’m struggling with that.” Personal (e.g., spending time with one’s children) and occupational (e.g., paperwork) time pressures also stood in the way:

As teachers, we all want to do [our best] and meet our expectations. And the thing is that we all–as much as we tell ourselves it’s not doable–we want to do it all [work and self-care]. That’s how we’re built. […] [W]e know we can’t, but yet it’s that constant battle within.

Teachers’ desire to bring their best effort to teaching is stifled by unrealistic workloads, which contribute to feelings of guilt about self-prioritization. Lastly, early career teachers and those new to campus were simply unaware of the mental health resources provided to them: “I literally didn’t know [mental health clinic] was available to teachers. […] I think it took like a good 2–3 weeks [before] I was like, ‘what is [mental health clinic name]?”’ Though only mentioned a few times, service awareness is a basic requirement for service access (Penchansky, 1977), which is especially important for early career teachers.

#### Ways Personal Factors Facilitate Stress Management

Teachers offered up examples of how they manage stress without guilt and commented on how administrators could facilitate service awareness. Some teachers shared how they think about taking time for themselves:

I’m at a point where I have been able to put the work in to not feel guilty about that [using planning periods for yoga]. […] When it comes to [self-care during] the workday, if you want me to be my best teacher and if I want my kids [students] to not be yelled at all the time, then I need that time.

The other piece of personal support, awareness of available services, focused on the new teacher experience: “I would have appreciated, like when I first came to [school name], an explanation. Like these are our services.” The low frequency of personal facilitators for stress management amplifies the need to address personal barriers to formal and informal SMIs. Self-care descriptive norm feedback–sharing how common self-care practices are among faculty and indicating the positive benefits associated with increased self-care (Schultz et al., 2018)–may help teachers feel less guilty about taking time for themselves. These relatively small steps may help motivate teachers to engage in formal or informal SMIs without guilt. Not surprisingly, environmental factors were also discussed regularly and included a variety of considerations.

### Environmental Stress Management Barriers and Facilitators

Teachers frequently discussed external circumstances that created barriers to and supported stress management and overall wellbeing. Five barrier themes emerged: (a) unconducive social climate, which included mental health stigma and lack of peer support; (b) inconsistent administrative support, which included siding with parents, unclear expectations, poor follow through, and mixed messages about self-care; (c) restrictive district policy, which included time consuming professional development and pressure to cover classes; (d) inconvenient service characteristics, which included location and hour constraints, capacity issues, excessive intakes processes, and prohibitive costs; and (e) campus facility issues, such as inadequate privacy and widespread campus layouts. The ways environmental contexts support stress management often mirrored barrier themes.

### Barriers to Stress Management Due to Social Climate

Two social barriers were often mentioned: stigma around help-seeking and lack of teaching teams. Participants spoke of mental health stigma in schools: “There’s this compound of, on top of the normal stigma, that maybe you’re not fit for the job if you show any indication of having mental health concerns.” Teachers also noted how social support is undermined when teaching teams are dissolved or absent in the school community: “I miss it [teaming] a lot. […] At least I had a group of teachers who supported me. Now, I don’t get as much support because they are all together except for me; […] I’m in the science wing.” Teaming was often discussed as supportive of wellbeing.

#### Ways Social Climate Facilitate Stress Management

Teaching teams and mentorship were two ways teachers experienced supportive campus climates. Teams provided support through collective boundary-setting, like ending the workday at 3:30 p.m. Team boundaries were especially helpful when working from home during COVID-19 shutdowns. Teams also provided classroom coverage:

My team and I were so close that I never really felt alone because I always had three people who had my back. If I had to go to the bathroom, if my mom called me or something bad happened in [city name], someone could cover my class.

Near-peer support for early-career teachers was also helpful: “I wouldn’t have been able to survive that [first] year at that crazy school if I didn’t have the teachers that had been there 20 years showing me the ropes.” Social connectedness can decrease stress and contribute to occupational wellbeing (Bermejo-Toro et al., 2016), so it is not surprising that teaching teams help teachers meet a variety of workplace demands. Interventions that address the specific connotations of mental health issues for teachers should also consider methods for reaching teachers. The support that often forms in teaching teams may provide a safe space to discuss mental health struggles and stigma.

### Barriers to Stress Management Due to School Administration

Inconsistent administrative support took many shapes for teachers: frequent siding with parents, inconsistent expectations between administrators, poor implementation of teacher resources, and insincere messages about self-care. A tension was felt between administrators’ intentions and actions:

It’s constant; you’re getting a mixed message. Take care of yourself. Make time. But then at the same time, ‘I’m going to keep adding more on without taking anything off. I recognize that I’m putting more on without taking anything off, but that’s what it is.’

Also included in this frequently discussed theme was the lack of procedures for handling personal emergencies: “There’s no time to process. There’s no structure to support being able to leave the classroom when crises come up.” On the other hand, when administrative support was discussed, it was viewed as immensely helpful.

### Ways Administration Facilitate Teachers’ Stress Management

Teachers valued administrators who demonstrated respect for teachers. These included turning a blind eye to district policies (e.g., signing in and out during breaks) that undermined teachers’ autonomy (e.g., crossing the street to get coffee during lunch) and taking initiative on teacher concerns:

We have a new assistant principal who I’m very impressed with, so far. If I have an issue with a student, I can talk to him about that student without having to write up a referral or send a bunch of documentation to him. I can just go talk to him, and he has actually called the student in. Then, he gets back to me, so it’s not like it’s just gone out in space somewhere.

Efficient meetings and meaningful use of training time were also discussed: “I think one thing that recently helped [facilitate stress management] was when we had professional development and we got to either paint or walk or do a physical activity.” Taken together, genuine displays of respect and care from administrators can empower teachers to make decisions that reduce stress and support wellbeing. Administrators who view teachers as capable professionals are more likely to create a supportive environment.

### Barriers to Stress Management Due to District Procedures

District-wide training requirements and pressure to cover for other teachers’ during planning periods (due to a district-wide substitute shortage) hindered campus-level wellbeing: “I wouldn’t say that it’s a campus administration [problem]. I think it’s more of the district. I think this campus administration has been phenomenal, amazing. […] I think what […] they make us do, is what they’re being told.” Teachers also spoke about challenges accessing district-provided services; in these instances, it was often difficult to determine whether the district or service or both were responsible for the challenge. These barriers appeared to be most directly connected to service characteristics, and thus were grouped as such. However, as participants pointed out, the district’s role in shaping the accessibility of services (e.g., EAP counseling session limits).

### Ways District Benefits Facilitate Stress Management

District-provided discounts for products and services (e.g., movie passes, gym memberships) came up a few times: “I’ve been using […] the Calm App. They [the district] offer it, it’s paid, but if you have a [district name] email, then it’s free. And it’s actually really awesome.” While district-provided services were often valued, district initiatives, if not moderated by administrators, often felt like additional time commitments. Perhaps district leaders can give greater discretion to school administrators for adopting and implementing initiatives. Interestingly, most participants were unaware of basic employee benefits; diversified communication channels may help with awareness.

### Barriers to Stress Management Due to Service Characteristics

Teachers found inconvenient locations and hours, limited client capacity, time-consuming intake procedures, and high costs to hinder help-seeking: “It’s [EAP counseling is] not easy to schedule. It’s not like we can schedule something at one o’clock during the weekday, [so] you’re limited to Saturday sessions or evenings and those fill up really quickly.” Participants frequently encountered these issues when seeking mental health services, but physical therapy and gyms were also mentioned. As was articulated previously, service accessibility issues were often connected to district decisions (e.g., employee gym inconveniently located at the district office).

### Ways Service Characteristics Facilitate Stress Management

Teachers frequently discussed the benefits of co-located mental health clinics, minimal intake procedures, and low-cost services. All campuses included in this study had an on-site, non-district run mental health clinic. The limited spaces in many of these clinics and exhaustive intake procedures were drawbacks for some, but their nesting within campus culture was seen as a significant benefit by others, “I felt like I would be understood. […] There’s a familiarity by being at [school] that they can see what it’s like. I felt like I was talking to someone who I didn’t have to give a huge backstory to.” While EAP hours and session limits came up as barriers, the connection to employee benefits was helpful: “I think [intake] is easier with the EAP probably because we’re already in the system, and it just makes it easier. Technically, the paperwork is already there […] for EAP. Insurance. Everything.” There appear to be sacrifices with the two dominant approaches to providing mental health support to teachers: (a) on-site, non-affiliated clinics provide a sense of familiarity and scheduling convenience but require extensive in-take procedures and often carry a waitlist, (b) EAPs can be helpful for crisis situations or short-term therapy and have streamlined intake procedures but fail to provide continued support and require a commute. The rapid movement to telepsychology (due to COVID-19) may open new opportunities for online counseling. Policy makers should consider locations, scheduling, and community connection when deciding on mental health services.

### Barriers to Stress Management Due to Campus Facilities

Teachers, who participated mostly in-person, described inadequate campus facilities for unwinding during the day, campus layouts that hindered social connection among faculty, and long walking distances that cut into break times. The spaces that were available were not suitable for destressing: “There’s a designated spot for the teachers to go and work together. […] It is glass [windows], so people walking by is distracting. […] They [kids] see you and they want to say ‘hi.’ […] It doesn’t feel very relaxing.” One school was recently constructed with two stories and two wings; when teachers moved to this new building, they noticed it was harder to stay connected with colleagues. Widespread campuses also made it difficult to get decent breaks, “I don’t see the people upstairs unless we happen to cross each other in the morning or I’m coming back from lunch. […] It’s not like, ‘hey we can chat and talk?’ because the school is so widespread.” Teachers who were teaching from home discussed the challenges of separating work and home life: Breaks were often filled with chores around the house. Some teachers shared helpful ways they used campus facilities.

### Ways Campus Facilities Enable Stress Management

Space restrictions were less of an issue for teachers who did not have to share a classroom: “I have actually cleaned out an area in my office where I go [to meditate], and I say between this time in this time, I am not available.” Others described how dedicated teacher rooms fostered comradery:

There was a teacher room and the administration did not go in. […] Subs were not in there. Teachers could, for [lack of a] better word, bitch to each other. […] It was a safe kind of place, what was said in the teachers room did not go out of the teachers room.

Colleague connection and boundaries are easier to support when designated spaces are available. As schools plan for reopening, administrators would be wise to consider how teachers will be provided space to take breaks in a safe yet meaningful way. Teachers discussed environmental and personal factors with the greatest depth and clarity. However, occasional references to behavioral patterns did emerge, and are considered next.

### Barriers to Stress Management Due to Behavioral Factors

Ways in which previous behaviors influenced teachers’ ability to manage stress rarely came up. The coding team often found it difficult to tease apart behavioral and cognitive influences: It was hard to tell whether views about stress management (e.g., “self-care is selfish”) or a particular practice (e.g., emotional suppression) sustained teachers’ challenges to wellbeing. The triadic reciprocal causation model sheds light on these challenges since the theory assumes that thoughts and behaviors influence each other as they shape psychosocial functioning (Bandura, 1986). For behavioral barriers, participants discussed patterns of suppressing emotions on the job:

At other jobs, most of the people that I know, they can get upset and go to their workroom or go home. That’s not what we do here. […] We’re used to sucking it up and taking it in, and then letting it out later. At that [later] time, we’re probably not going to go seek any assistance because we haven’t even been able to deal with that at that minute.

Participants also spoke about how disappointing outcomes from personal efforts, like failed workout routines and campus-change efforts, hindered future engagement, “You try to change things yourself and to maybe offer solutions or ideas [at school], and […] there doesn’t seem to be change. So I kind of figure, like, well, what’s the point?” The few accounts clearly associated with behavior reinforcement were strongly connected to broader structures of the school community that made positive outcomes much less likely. On the flip side, one example of how positive outcomes can reinforce future behavior emerged.

### Ways Behavioral Patterns Facilitate Stress Management

For one teacher, setting boundaries around breaks led to a shift in beliefs around taking time for themself and reinforced this behavior, “[P]eople come and hunt me down; and I’m sitting there eating […]. I’ve learned to say, ‘I need to have this 30 min to myself every day’ and say, ‘I can’t help you right now’.” Overall, the ways behaviors influence stress management were rarely discussed. This is likely because teachers were not probed to think specifically about behavioral influences. That said, reinforcement of teachers’ efforts to care for themselves seems important to consider when attempting individual-organizational change. Messaging about self-care needs to be reinforced otherwise the message may lose its power.

Reciprocal relationships were frequently encountered between personal and environmental factors. Similar patterns of connectedness emerged for desired resources.

### Desired Resources

**FIGURE 2:**
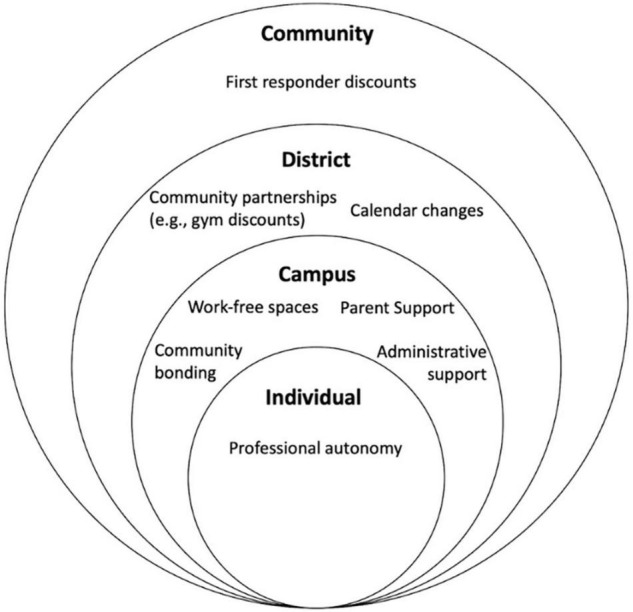
Teachers’ desired resources for well-being are shown within a socioecological framework.

The second goal of this study was to identify resources teachers felt would support stress management. Four emergent domains were modeled after McLeroy et al.’s (1988) ecological model of health behavior: individual, campus, district, and community level resources (see Figure 2 for a visual representation). The model, which belongs to a family of socioecological models, asserts that behavior is nested within everchanging spheres of influence (i.e., intrapersonal, interpersonal, institutional, community, and policy; McLeroy et al., 1988). From this view, the emergence of mostly interpersonal and institutional resource recommendations is not surprising: teachers’ stress management behaviors exist within a highly social and structured work environment.

#### Desired Resources at the Individual Level

Teachers desired greater control over their time usage: “Why don’t they [administrators] just let me decide what to do with my time? […] Let me manage it.” Ways administrators can support time management are discussed at length in campus resources.

#### Desired Resources on Campus

Teachers had the most to say about improving resources at the campus level. Topics included interpersonal methods for improving social support (i.e., from each other, administrators, and parents) and creation of dedicated destressing spaces (i.e., work-free zones).

#### Interpersonal Resources

Teachers desired stronger connections with colleagues. Social activities, group follow-through on decisions, shared campus pets, and opportunities for collective discussion-making were recommended: “I think that that [discussion] is a huge piece that is missing in this [school] family. The family needs to talk.” Support from school administration was also important, “I would like [administrators] to say, ‘Oh, yes, you absolutely are a professional. You are a teacher, and you have all of these skills, and I trust that you’re going to do these things.” Teachers also recommended administrators build in time for self-care both in the daily schedule (e.g., give middle schoolers recess to allow for longer lunch periods) and through professional development: “Please value our time and don’t just have us sit and go through a process of something unless it’s meaningful and it’s going to enhance our teaching […] or feed our soul.” Nearly all recommendations for improving administrative practice and policy related to increased autonomy. Administrators need to consider the underlying message sent to teachers when implementing new policy or actively providing stress management resources.

Although infrequent, teachers wanted to see greater parent support on campus. Examples included providing meals and organizing stress relieving activities, “The parents in the community come and do things for the teachers [every Friday]—they feed them and do all these little workshops. The teachers could go if they want to, or they could just work in their rooms.” Recommendations for greater parent involvement often included a desire for increased connectedness among the school community, including parents.

#### Facility Resources

Teachers frequently wanted on-campus relaxation spaces with soft lighting, inviting furniture, and policies prohibiting work questions:

There needs to be a place where work is not expected to happen. […] People know when I walk into that space, we’re not having a work conversation. I didn’t go there to find you to ask you to do an extra favor for me.

Teachers often acknowledged the importance of relaxation spaces being designated for self-care or stress management and being separate from teacher workrooms or offices.

#### Desired Resources at the District Level

Two district-level resources emerged: calendar changes and community partnerships. Changes to the district calendar, such a 4-day work week or year-round schooling, were recommended ways of creating breathing room in teachers’ schedules:

The school that I know that does it [year-round schooling], they have a week off at the end of every grading cycle. They have 2 weeks off at Christmas. They get spring break, and they have 6 or 8 weeks off in the summer. They still have the same amount of time that we have. It’s just built differently into the school year. I just know that we go from ending one 6 weeks to starting another 6 weeks without time to even plan the next 6 weeks.

A range of non-traditional school schedules have been introduced over the past decade or so, in part due to amazingly underfunded district budgets (i.e., 4-day work weeks). Reimagining public education could include discussion of academic calendar changes.

Teachers also suggested district partnerships with local businesses to make community resources more affordable: “It’d be wonderful if there was a way to network with a gym where teachers get a discount […] that’s just part of your package for being an employee with [district name].” Surveying teachers on the services they want and need, while also considering accessibility, could improve the impact of district-level SMIs. Additionally, desired campus-level changes will need significant support from school districts. This connection is key to successful multilevel intervention.

#### Desired Resources at the Community Level

Although rare, a few participants voiced their desire for community companies to show appreciation for teachers through discounts on wellbeing services and other products:

We get kicked and spit on and cussed at and have to deal with stuff all the time. I think that we should have the same kind of appreciation that first responders do. […] Home Depot and Lowes and Planet Fitness, all these places offer [discounts] for first responders, we should be considered first responders. We are. We call them.

Interestingly, this comment was made in early March of 2020. Shortly after, a few local and national companies announced discounts for teachers (due to COVID-19). Time will tell if these benefits will last or change public perception of teachers. Teachers’ recommendations across socioecological levels highlight the need for multilevel SMI.

### Theme Discussion

Barriers to and facilitators for teachers’ engagement in SMIs, formal and informal, fell into three domains, consistent with Bandura’s (1986) triadic reciprocal causation model: personal, environmental, and behavioral. Personal and environmental barriers were the most frequently discussed domains. Notably, self-prioritization beliefs–thoughts and feelings about prioritizing ones’ own self-care above work duties, student needs, or family obligations–were the themes most frequently discussed. Many teachers felt their many other responsibilities prohibited engagement in formal and informal SMIs. Teachers often noted the environmental barriers that contributed to and maintained teachers’ negative thoughts and feelings on self-prioritization. This is consistent with the bidirectional relationship between elements in the triadic reciprocal causation model (Bandura, 1986). Personal and environmental barriers and facilitators, were often described in terms of their influence on one another (e.g., teachers being required to cover for absent coworkers during planning periods perpetuated teachers’ belief that their time was not respected by district and school administrators). The interplay between domains may help explain the discrepancy in SMI effectiveness between teachers (Iancu et al., 2017) and professionals as a whole (Richardson and Rothstein, 2008): While many of the most effective SMIs contain elements of cognitive reframing (in cognitive-behavioral and mindfulness-based programs; Iancu et al., 2017), the skills taught may be especially hard for teachers to enact as their social and physical work environments limit stress management autonomy in ways not experienced by most other professionals (Guo and Wang, 2021). For example, teachers in the United States are the least likely group of professionals to report their opinions matter at work (Hodges, 2018), suggesting teachers need to feel heard and empowered in their work environments before they are able to engage with stress management in a meaningful way. Teachers did provide valuable ideas for improving resources supportive of teacher wellbeing, including increased autonomy in matters of time and self-care.

The desired resources shared by teachers centered mostly on campus-level changes. The most common suggestions were to encourage social connection within faculty and support from administrators and parents. This need was highlighted by teachers experiencing community shut-downs due to the COVID-19 pandemic and adjusting to virtual instruction from home. As teachers are now back in the classroom, for the most part, social connection (in a way that feels safe) is likely a much-desired resource for teachers who often felt isolated during the pandemic-related shut-downs (Eyal et al., 2022*;* Gearhart et al., 2021). Increasing opportunities for and shows of social support may also address the social culture and administrative support barriers that were often connected to an unwillingness among teachers to show mental health vulnerabilities. Facilities conducive to wellbeing were also frequently discussed. Teachers felt designated spaces for relaxing support a culture of wellbeing and individual efforts to destress during the day. This recommendation was of high importance to the teachers at the one high school included in this study, possibly because these teachers lack such a space and found it difficult to ask students and other faculty to leave their classrooms during off periods and lunches.

### Implications for Teacher Support

The frequently discussed negative self-prioritization beliefs illuminate one potential reason for the small effect sizes for individual-level SMIs with teachers: Simply because resources are available, does not mean teachers feel comfortable accessing them, particularly those working in high need schools with scant resources for students. Shifting the campus culture to one that values and supports wellbeing for teachers (and likely all school community members) seems necessary for effective stress management among teachers. Specific to teachers, designating professional time for stress management can demonstrate administrative support for teachers’ wellbeing, and campus-wide messaging about taking time to connect with colleagues and the benefits of prioritizing self-care may facilitate meaningful and sustained engagement with formal and informal SMIs. Additionally, peer or health coaches may help circumvent setbacks due to negative views about stress management. Lastly, campus climate surveys could be used to directly ask teachers about the availability of SMI resources as well as teachers’ comfort levels in accessing such resources.

The high frequency and diversity of environmental barriers to SMIs and campus-level desired resources align with Greenberg et al.’s (2016) call for the study and implementation of individual-organizational and organizational interventions. The specifics of these SMIs should be developed with campus-level contexts and teachers’ needs in mind. Informal socializing and spaces to connect over non-school matters can help to deepen an already supportive campus community. These social opportunities are especially important as teachers recover from the especially trying academic years during the COVID-19 pandemic.

## Limitations

The current study benefited from teachers’ voices across public school settings and faculty positions. However, some limitations are important to note. First, data was collected through focus groups conducted with teachers from shared campuses. This shared experience allowed for participants to build off one another’s experiences but may have also limited participants’ willingness to share fully about issues of mental health or violations of school policy. Individual interviews or other private data collection processes may have changed how often stigmatized behaviors were discussed. Additionally, participants worked for the same large, public school district, and thus shared a sociopolitical context: District and community recommendations may look different in other contexts (e.g., rural areas, charter schools). The majority of teachers in this study were established in their field (i.e., more than 5 years of experience). There may be additional barriers to consider for early career teachers (Antoniou et al., 2006; Klassen and Chiu, 2010).

Lastly, data was collected during the emerging months of the COVID-19 pandemic; the situation was changing daily, and the widespread view was to take the situation day-by-day, week-by-week. It seemed teachers simply were not thinking about virtual schooling as a “new normal” but instead a temporary condition. This might explain why the conditions of virtual teaching and school shutdowns, while present in the data, did not emerge as unique themes. It is likely that some of the barriers and recommendations (e.g., designated teacher relaxation spaces) may need to look somewhat different than recommended by participants. Other recommendations may be more salient for teachers as in-person teaching has resumed in the United States and many other areas (e.g., connecting with colleagues). Despite these limitations, a wide range of views were captured.

## Conclusion

This study provides guideposts for reducing teachers’ stress across the individual-organizational continuum. Findings point to campus-level assessment of culture and intra- and interpersonal support. Incorporation of teachers in this process not only supports their much-needed autonomy, but also allows for targeted intervention. Findings emphasize the need for multilevel change, as barriers to stress management often included person-environment interaction. While the results suggest avenues for removing barriers to individual-level SMI engagement, individual intervention is limited without campus and district change. Policy changes that support teacher autonomy are important for removing barriers to teacher wellbeing.

## Data Availability Statement

The raw data supporting the conclusions of this article will be made available by the authors, without undue reservation.

## Ethics Statement

The studies involving human participants were reviewed and approved by Institutional Review Board, University of Texas at Austin. The patients/participants provided their written informed consent to participate in this study.

## Author Contributions

CG and CM contributed to conception and design of the study. CG conducted focus groups, oversaw data analysis, and wrote the first draft of the manuscript. CG and MB participated in the qualitative coding of data. MB and CM wrote sections of the manuscript. All authors contributed to manuscript revision, read, and approved the submitted version.

## Conflict of Interest

The authors declare that the research was conducted in the absence of any commercial or financial relationships that could be construed as a potential conflict of interest.

## Publisher’s Note

All claims expressed in this article are solely those of the authors and do not necessarily represent those of their affiliated organizations, or those of the publisher, the editors and the reviewers. Any product that may be evaluated in this article, or claim that may be made by its manufacturer, is not guaranteed or endorsed by the publisher.
